# Protective Effect of Toll-like Receptor 4 in Pulmonary Vaccinia Infection

**DOI:** 10.1371/journal.ppat.1000153

**Published:** 2008-09-19

**Authors:** Martha A. Hutchens, Kathryn E. Luker, Joanne Sonstein, Gabriel Núñez, Jeffrey L. Curtis, Gary D. Luker

**Affiliations:** 1 Graduate Program in Immunology, University of Michigan Medical School, Ann Arbor, Michigan, United States of America; 2 Department of Radiology, University of Michigan Medical School, Ann Arbor, Michigan, United States of America; 3 Division of Pulmonary and Critical Care Medicine, Department of Internal Medicine, University of Michigan Medical School, Ann Arbor, Michigan, United States of America; 4 Department of Pathology, University of Michigan Medical School, Ann Arbor, Michigan, United States of America; 5 Comprehensive Cancer Center, University of Michigan Medical School, Ann Arbor, Michigan, United States of America; 6 Department of Veterans Affairs Health System, University of Michigan Medical School, Ann Arbor, Michigan, United States of America; 7 Department of Microbiology and Immunology, University of Michigan Medical School, Ann Arbor, Michigan, United States of America; Saint Louis University, United States of America

## Abstract

Innate immune responses are essential for controlling poxvirus infection. The threat of a bioterrorist attack using *Variola major*, the smallpox virus, or zoonotic transmission of other poxviruses has renewed interest in understanding interactions between these viruses and their hosts. We recently determined that TLR3 regulates a detrimental innate immune response that enhances replication, morbidity, and mortality in mice in response to vaccinia virus, a model pathogen for studies of poxviruses. To further investigate Toll-like receptor signaling in vaccinia infection, we first focused on TRIF, the only known adapter protein for TLR3. Unexpectedly, bioluminescence imaging showed that mice lacking TRIF are more susceptible to vaccinia infection than wild-type mice. We then focused on TLR4, the other Toll-like receptor that signals through TRIF. Following respiratory infection with vaccinia, mice lacking TLR4 signaling had greater viral replication, hypothermia, and mortality than control animals. The mechanism of TLR4-mediated protection was not due to increased release of proinflammatory cytokines or changes in total numbers of immune cells recruited to the lung. Challenge of primary bone marrow macrophages isolated from TLR4 mutant and control mice suggested that TLR4 recognizes a viral ligand rather than an endogenous ligand. These data establish that TLR4 mediates a protective innate immune response against vaccinia virus, which informs development of new vaccines and therapeutic agents targeted against poxviruses.

## Author Summary

Toll-like receptors are a class of transmembrane proteins that detect the presence of infectious organisms and activate host innate and adaptive immune responses. Vaccinia virus is the prototypic poxvirus, and it is used as both a model and a vaccine for the virus that causes smallpox. We recently reported that Toll-like receptor 3 (TLR3), which recognizes double-stranded RNA, acts in vaccinia infection in a way that is detrimental to the host. TLR3 relays its signal to the nucleus using the adaptor protein TRIF. In this paper, we report that mice lacking TRIF are more susceptible to vaccinia infection than wild-type controls. TLR4 also uses TRIF to relay its signals. We report our findings that TLR4 has a protective effect in vaccinia infection. Mice with a nonfunctional mutant version of TLR4 are more susceptible to vaccinia infection than wild-type controls. The protection that TLR4 affords is not due to effects on secretion of proinflammatory cytokines or type I interferon, and the receptor also does not uniquely regulate recruitment of white blood cells to the site of infection. Rather, TLR4 recognizes a molecule in or on vaccinia virus to bring about a protective response that may be due to an ability to diminish the degree of inflammation caused by vaccinia infection.

## Introduction

In 1980, the World Health Organization declared that smallpox had been eliminated as a human disease [1]. Nevertheless, potential bioterrorist release of *Variola major*, the causative agent for smallpox, and human infection with monkeypox or other zoonotic orthopoxviruses has heightened interest in this family of viruses [2]. *Variola major* is particularly feared as a bioterrorism agent because of the high rate of transmission and up to 30% mortality caused by smallpox [3]. Fatal cases of smallpox were characterized by clinical findings similar to septic shock, likely mediated by the host inflammatory response to infection. However, molecules and signaling pathways that initiate and control protective and detrimental immune responses to *Variola major* remain poorly defined. Identifying molecular determinants of the innate immune response to poxviruses is critical to understanding pathogenesis of poxvirus infections and developing better therapies to prevent or ameliorate the sepsis-like disease manifestations. This knowledge also may lead to development of a safer smallpox vaccine that eliminates the high risk of severe, life-threatening complications associated with the current live, attenuated vaccinia virus vaccine. Improved understanding of the innate immune response to poxviruses will have benefits beyond advancing new vaccines and therapies to prevent and treat infection. Vaccinia virus is being investigated as a gene delivery, oncolytic, or immunizing vector for a wide variety of diseases, including cancer, HIV and malaria [4]–[8]. Greater knowledge of normal host-pathogen interactions will enable more efficient targeting and efficacy of these vectors in patients. Finally, insights gained from studying pulmonary infection with poxviruses are expected to inform research on protective and harmful aspects of host immunity to other respiratory pathogens.

Toll-like receptors (TLRs) have emerged as key molecules in initiating innate immune responses to a variety of different pathogens, and these receptors also regulate subsequent adaptive immune responses to infection. TLRs recognize defined molecular patterns associated with various pathogens, including bacteria, fungi, and viruses. In vitro studies have identified canonical ligands for different TLR family members, such as double-stranded RNA for TLR3 and bacterial lipopolysaccharide (LPS) for TLR4. However, recent studies suggest that TLRs may respond to a broader range of molecular patterns. For example, while TLR4-dependent recognition of LPS is well-established as a central regulator of effective host immunity to bacterial pathogens, TLR4 also may signal in response to a wide variety of endogenous ligands, such as heat shock proteins [9],[10]. TLR4 also may respond to some viral proteins, and TLR4-dependent signaling may be necessary to limit viral replication and disease morbidity in vivo [11],[12]. These studies emphasize that functions of TLRs in host immunity may extend to pathogens that do not carry known ligands for specific receptors, particularly as TLRs respond to infections in living animals.

We recently established that TLR3 controls a detrimental innate immune response to pulmonary infection with vaccinia virus, a model virus for studies of orthopoxviruses [13]. Compared with wild-type mice, mice lacking TLR3 (TLR3^−/−^) had reduced viral replication and were protected against disease morbidity and mortality. Adverse effects of TLR3 signaling were caused in part by an excessive inflammatory response to infection. To further investigate TLR3 in poxvirus infection, we initially focused on functions of TIR domain-containing adapter inducing interferon-β (TRIF), the only known downstream adapter molecule for TLR3. Unexpectedly, mice lacking TRIF (TRIF^−/−^) did not reproduce protective effects of deleting TLR3, but TRIF^−/−^ was more susceptible to vaccinia infection. These data prompted us to analyze functions of TLR4, the only other TLR known to signal through TRIF, in response to respiratory infection with vaccinia virus. We determined that TLR4 signaling protects mice against vaccinia infection, limiting viral replication and local inflammation.

## Results

### TRIF^−/−^ mice have a distinct phenotype from TLR3^−/−^ mice

We recently reported that TLR3^−/−^ mice are protected from pulmonary vaccinia infection compared to wild type C57BL/6 controls [13]. The only known adaptor molecule for TLR3 is TRIF, so TLR3 is thought to signal exclusively through TRIF to control secretion of type I interferons and pro-inflammatory cytokines [14]. Because of this direct TLR3 to TRIF signaling pathway, we hypothesized that TRIF**^−/−^** mice would be protected against vaccinia infection, similar to TLR3^−/−^ mice.

We infected TRIF^−/−^ and wild-type C57BL/6 mice with 1×10^4^ pfu Vac-GFL intransally (i.n.) to reproduce the natural respiratory route of infection with *Variola major*. Vac-GFL is a recombinant vaccinia virus that expresses a reporter protein comprised of GFP fused to firefly luciferase [13], which allows viral replication and dissemination to be quantified in living mice using bioluminescence imaging. We previously established that in vivo measurements of bioluminescence from Vac-GFL correlate directly with viral titers in a defined organ or tissue [15]. Bioluminescence imaging was performed daily to monitor replication of vaccinia virus, and weight loss was used as marker for systemic severity of disease.

**Figure 1:**
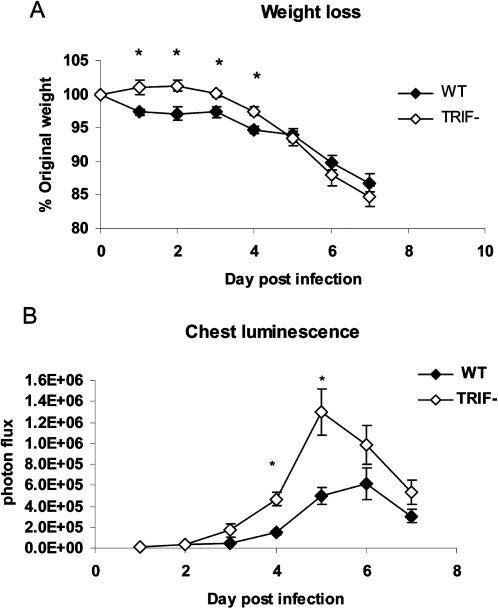
TRIF^−/−^ mice are more susceptible to vaccinia infection than WT. TRIF^−/−^ and WT BL/6 mice were infected with 1×10^4^ pfu Vac-FL. (A) Weight loss, expressed as percent of initial weight. *p<0.05. (B) Chest luminescence, expressed as photon flux. Error bars denote SEM.

Unexpectedly, susceptibility of TRIF^−/−^ mice to vaccinia infection was distinct from that of the TLR3^−/−^ mice. TRIF^−/−^ mice had less weight loss than wild-type mice on days 1–4 post-infection (*p*<0.05), which is similar to our published results for TLR3^−/−^ versus wild-type mice, (Figure 1A). However, TRIF^−/−^ mice differed from TLR3^−/−^ animals in that replication of Vac-GFL was greater in mice lacking TRIF, as quantified by region of interest analysis of head, chest, and abdomen sites on bioluminescence images. By area under the curve (AUC) analysis, TRIF^−/−^ mice had significantly greater luminescence in their chests (from lung infection) than wild-type mice (Figure 1B; *p*<0.01). These data indicate that a different and/or additional host molecule(s) controls responses to vaccinia in TRIF^−/−^ mice relative to those mediated solely by TLR3. This experiment continued until day 7 post-infection, when the animals were euthanized to obtain plasma and bronchoalveolar (BAL) fluid for quantification of cytokines. Levels of IL-6, IL-4, IFN-γ, MCP-1, TNF-α, and TGF-β were measured in these samples, but no significant differences were seen between the TRIF^−/−^ and WT mice (data not shown).

### TLR4 confers protection in pulmonary vaccinia infection

We hypothesized that TLR4, the only other Toll-like receptor known to signal through TRIF, may control differing host responses to vaccinia in TLR3^−/−^ versus TRIF^−/−^ mice. TLR4 is reported to limit replication of a limited number of viruses [11],[16], although functions of this receptor in vaccinia infection have not been established. To investigate TLR4 in host defense against vaccinia virus, we used C3H/HeJ mice, which have a point mutation in the cytoplasmic region of TLR4 that renders them unresponsive to LPS [17]. As controls, we used C3HeB/FeJ mice, which have normal, functional TLR4. C3HeB/FeJ mice are genetically similar to C3H/HeJ mice and are well-established as a control strain for experiments using C3H/HeJ animals [18]–[20].

**Figure 2:**
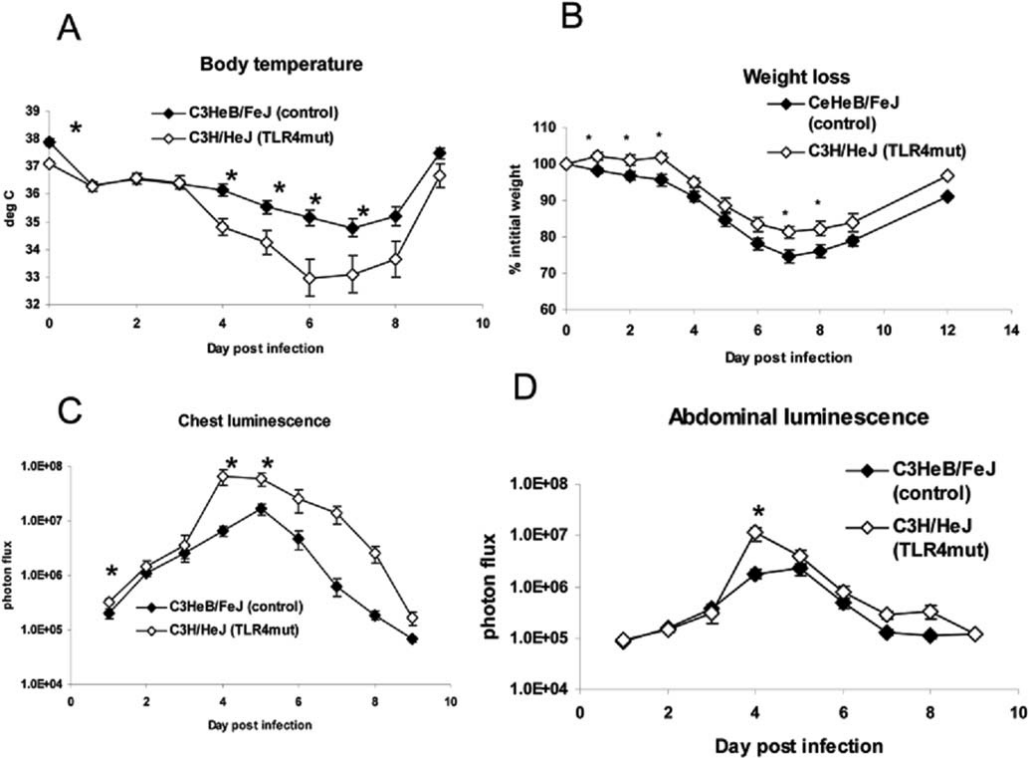
TLR4 mutant mice are more susceptible to vaccinia than controls. C3HeB/FeJ and C3H/HeJ mice were infected with 1×10^4^ pfu Vac-GFL. (A) Body temperature. (B) Weight loss, expressed as percent of initial weight; (C) Chest luminescence. (D) Abdominal luminescence, expressed as photon flux. *p<0.05. Error bars denote SEM.

We infected C3H/HeJ and C3HeB/FeJ mice with 1×10^4^ pfu Vac-GFL. Systemic effects of disease were monitored by weight loss and rectal temperature, while viral replication and spread were assessed with bioluminescence imaging. While both strains of mice lost body temperature in response to vaccinia infection [21], temperatures decreased to a greater extent over the course of the infection in C3H/HeJ (TLR4 mutant) mice relative to C3HeB/FeJ (Figure 2A). C3H/HeJ mice had a slightly lower temperature than the controls prior to infection, but there were no differences between strains on days 1, 2, and 3 post-infection. Beginning on day 4, however, rectal temperatures were significantly lower in C3H/HeJ mice, reaching a mean temperature of 33°C on day 6 post-infection (*p*<0.05). In contrast, the lowest mean temperature recorded in C3HeB/FeJ mice was 34.7°C on day 7. C3H/HeJ mice had significantly lower temperatures than control C3HeB/FeJ mice on days 4–7 (*p*<0.05). As a second marker of disease severity, weight loss was monitored over the course of the disease (Figure 2B). Surprisingly, the TLR4 mutant mice lost slightly less weight than the controls with significant differences on days 1–3 and 7–8 post-infection (*p*<0.05). Although the pattern of the weight loss difference is opposite that of the body temperature, it is consistent with the reduced weight loss observed in TLR3^−/−^ and TRIF^−/−^ mice compared with wild-type controls.

Bioluminescence imaging showed that C3H/HeJ (TLR4 mutant) mice had significantly greater viral replication in the chest region on days 1, 4, and 5 post-infection (*p*<0.05; Figure 2C). Light measured in the chest region of interest predominantly represents viral replication in the lung. C3H/HeJ mice also had significantly more Vac-GFL bioluminescence in the chest over the full course of the experiment as determined by AUC analysis. AUC values for bioluminescence were 1.61×10^8^ vs. 3.18×10^7^ for C3H/HeJ and C3HeB/FeJ mice, respectively (*p*<0.01). C3H/HeJ mice also had increased abdominal luminescence compared to control animals on day 4 (*p*<0.05) and over the course of the experiment by AUC analysis (Figure 2D). AUC values for photons produced in abdominal regions were 1.68×10^7^ and 5.21×10^6^ for C3H/HeJ and control C3HeB/FeJ mice, respectively (*p*<0.05). Bioluminescence in the head region did not differ between groups, and all mice recovered from infection (data not shown). Collectively, these data suggest that TLR4 limits respiratory infection and systemic spread of vaccinia virus.

**Figure 3:**
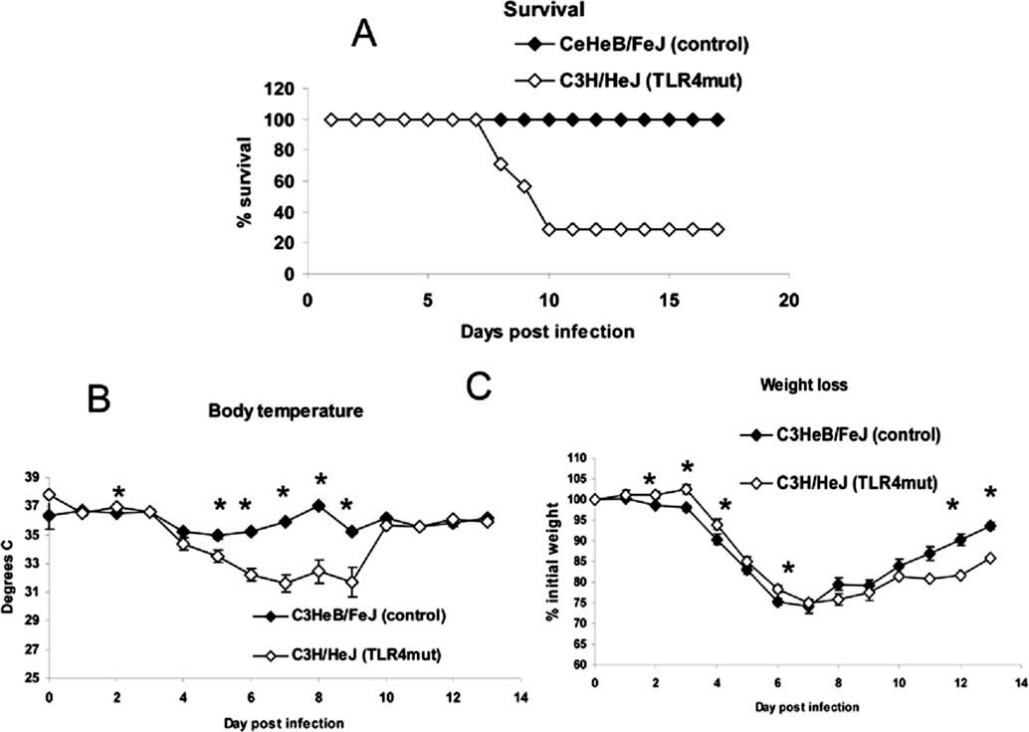
Increased susceptibility of TLR4 mutant mice is more pronounced at higher viral dose. C3HeB/FeJ and C3H/HeJ mice were infected with 5×10^5^ pfu Vac-GFL intranasally. (A) Survival curve, expressed as percentage of mice surviving. (B) Body temperature. (C) Weight loss, expressed as percentage of initial weight. *p<0.05. Error bars denote SEM.

To establish effects of TLR4 on survival, we infected C3H/HeJ and control C3HeB/FeJ mice with 5×10^5^ pfu Vac-GFL, a dose 1.5 logs higher than used previously. Using this inoculum, TLR4 mutant mice were clearly more susceptible to vaccinia infection. By day 10 post-infection, 70% of C3H/HeJ mice had died, while all control mice recovered from infection (Figure 3A). As in the previous experiment, loss of body temperature was measured as a sign of morbidity. C3H/HeJ mice were significantly more hypothermic than control C3HeB/FeJ mice on days 2 and 5–9 post-infection (*p*<0.05; Figure 3B). The rapid recovery of mean temperature in C3H/HeJ mice between days 9 and 10 is caused by death of the most hypothermic mice, while the surviving animals recovered temperature comparable to control C3HeB/FeJ mice. These data were consistent over 5 independent experiments. Weight loss also was monitored over the course of the disease. C3H/HeJ mice exhibited less weight loss than control C3HeB/FeJ animals over the first 7 d post-infection, and these differences were significant on days 2–4 and 6 (*p*<0.05; Figure 3C). The same trend was observed in two subsequent experiments. However, C3H/HeJ mice recovered weight more quickly than control C3HeB/FeJ animals on days 8–13, with significant differences observed on days 12 and 13 (*p*<0.05). Both body temperature and weight loss are reported to be regulated by cytokines, including IL-1, IL-6, and TNF-α, as part of the “sickness response” [22]. The discrepancy between these parameters during vaccinia infection suggests underlying differences in mechanisms and pathways that regulate these two global measures of disease. These data highlight limitations of using weight loss alone as a measure of disease severity in vaccinia infection.

**Figure 4:**
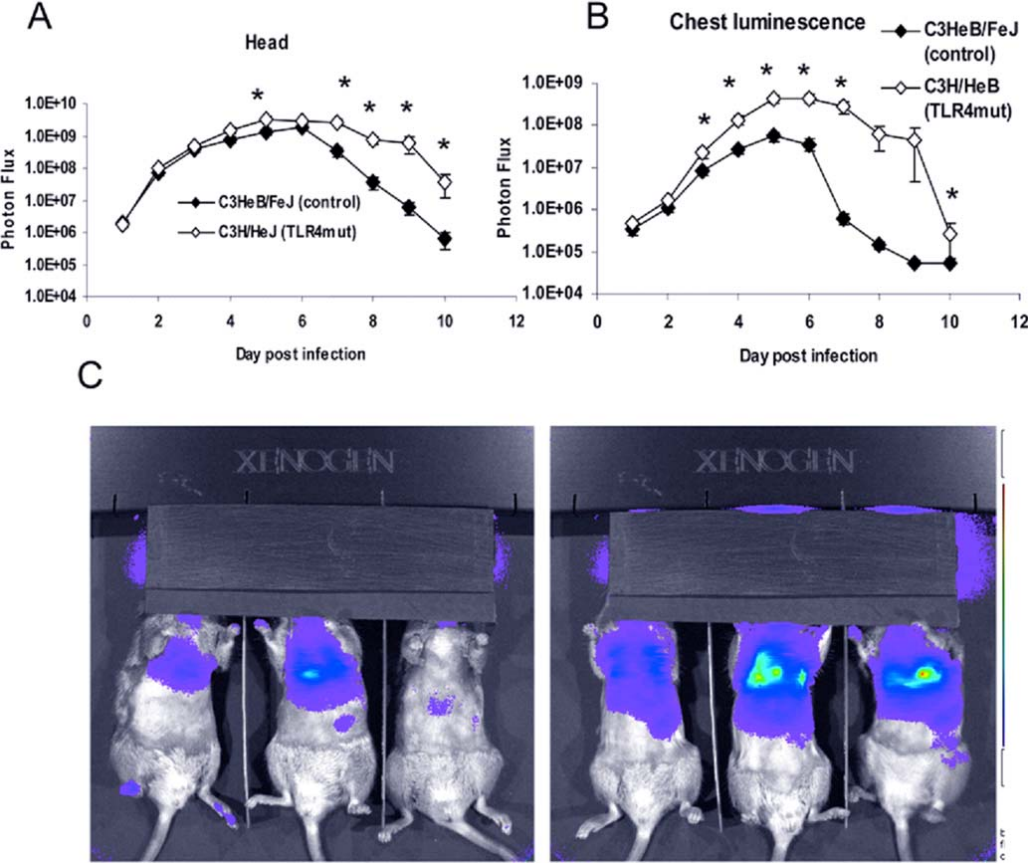
Increased viral replication in TLR4 mutant mice is more pronounced at higher viral dose. C3HeB/FeJ and C3H/HeJ mice were infected with 5×10^5^ pfu Vac-GFL intranasally. (A) Head luminescence. (B) Chest luminescence. (C) Representative chest images. C3HeB/FeJ (left) and C3H/HeJ (right), 30 s exposure, f-stop 1. Purple denotes lower luminescence intensity; red, higher luminescence intensity.*p<0.05. Error bars denote SEM.

**Figure 5:**
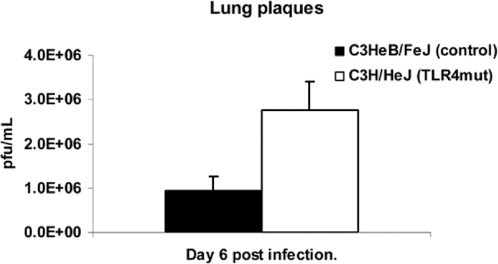
Increased viral titers in TLR4 mutant lungs. C3HeB/FeJ and C3H/HeJ mice were infected with 5×10^5^ pfu Vac-GFL intranasally. Lung viral titer expressed as pfu/mL; lungs harvested day 6 post-infection. *p<0.05. Error bars denote SEM.

**Figure 6:**
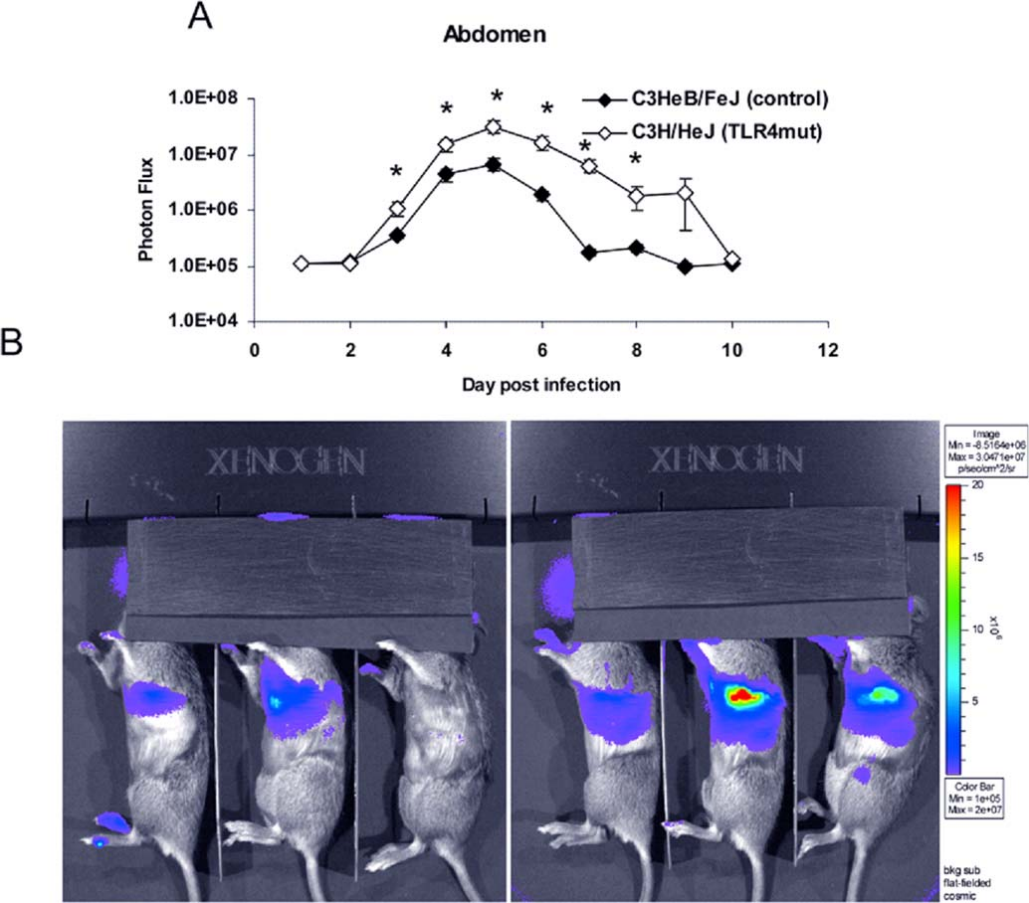
Increased viral replication in TLR4 mutant abdomens is more pronounced at higher viral dose. C3HeB/FeJ and C3H/HeJ mice were infected with 5×10^5^ pfu Vac-GFL intranasally. (A) Abdominal luminescence. (B) Representative images of splenic luminescence C3HeB/FeJ (left) and C3H/HeJ (right) mice, 30 s exposure, f-stop 1. Purple denotes lower luminescence intensity; red, higher luminescence intensity.*p<0.05. Error bars denote SEM.

With an inoculum of 5×10^5^ pfu Vac-GFL, differences in viral replication between genotypes were even more pronounced than in the earlier experiment. Bioluminescence from Vac-GFL was greater in the head region of C3H/HeJ mice compared with controls. Differences between strains were statistically significant over the latter part of infection on days 5 and 7–10 (*p*<0.05; Figure 4A). Over the course of the experiment, there was a trend for higher head bioluminescence in C3H/HeJ mice as determined by AUC analysis, although this difference was not statistically significant. Similarly, bioluminescence in the chests of TLR4 mutant mice was significantly increased over the controls (*p*<0.05) on days 3–7 and 10 post-infection (Figure 4B,C). At the peak of infection on day 6, the chest luminescence of the TLR4 mutant mice was 8-fold higher than that of the control mice. Moreover, the AUC for bioluminescence in C3H/HeJ TLR4 mutant mice was significantly greater than that for controls (7.22×10^8^ vs. 8.20×10^7^, respectively; *p*<0.05). Increased viral replication in lungs of C3H/HeJ was also confirmed by plaque assay (Figure 5). Finally, C3H/HeJ mice had greater systemic spread of the virus to the abdomen (Figure 6A and 6B). At the peak on day 5, the TLR4 mutant mice had 4.7-fold higher luminescence in the abdomen than wild-type controls. Differences between the two genotypes were significant on days 3–8 post-infection. The AUC of the abdominal luminescence in the C3H/HeJ mice was 4.39×10^7^ compared with 9.39×10^6^ in the control C3HeB/FeJ mice, respectively (*p*<0.05). These data extend our initial observations of increased viral replication and dissemination in mice lacking functional TLR4. Taken together, loss of functional TLR4 renders C3H mice more susceptible to pulmonary vaccinia infection, as measured by multiple parameters. Therefore, TLR4 must recognize some exogenous or endogenous ligand present in vaccinia infection.

To exclude the possibility of our results being affected by endotoxin contamination of our viral preparation, we infected RAW cells with Vac-GFL in the presence or absence of 10 µg/mL polymyxin B [23]. Levels of IL-6, TNF-α, and MCP-1 in the cell culture supernatants were assayed by ELISA. Adding polymyxin B did not affect levels of IL-6, MCP-1, or TNF-α in the supernatants of infected cells (data not shown), establishing that contaminating endotoxin did not affect our in vivo studies.

### Loss of TLR4 signaling does not abolish IFN-β production

**Figure 7:**
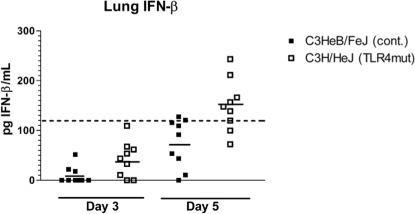
Lack of TLR4 does not impair IFN-β production. IFN-β concentrations in lung homogenate supernatants were measured by ELISA. Points represent individual mice. Dashed line represents lower limit of reliable detection on standard curve. Solid lines represent mean IFN-β concentration.

Type I interferons are essential to effective host defense against vaccinia infection [24]. TLR4 signaling results in production of Type I interferons through activation of transcription factors interferon regulatory factor 3 (IRF3) and NF-κB. To determine to what extent TLR4 regulates secretion of type I interferons during vaccinia infection, we measured concentrations of interferon-β (IFN-β) in lung tissue of C3H/HeJ and control mice. Mice were infected i.n. with 5×10^5^ pfu Vac-GFL, and lungs were harvested on days 3 and 5 post-infection. The day 3 time point is early in the course of infection, at the beginning, or just before, differences in luminescence and body temperature appear. Day 5 is near the peak of the infection where differences between TLR4 mutant and control mice are most pronounced. Lungs were homogenized and concentrations of IFN-β in supernatants were measured by ELISA. Levels of IFN-β were below the limit of reliable detection on day 3 in both groups of mice. On day 5 post-infection, IFN-β levels in 8 of 9 control mice remained below the limit of detection. On the other hand, six of 9 C3H/HeJ mice had IFN-β above the limit of reliable detection on day 5 (Figure 7). Therefore, C3H/HeJ mice are capable of producing IFN-β despite the lack of TLR4 signaling, showing redundancy in signaling pathways that activate a type I interferon response to vaccinia.

### Loss of TLR4 does not eliminate the inflammatory response to vaccinia in the lung

**Figure 8:**
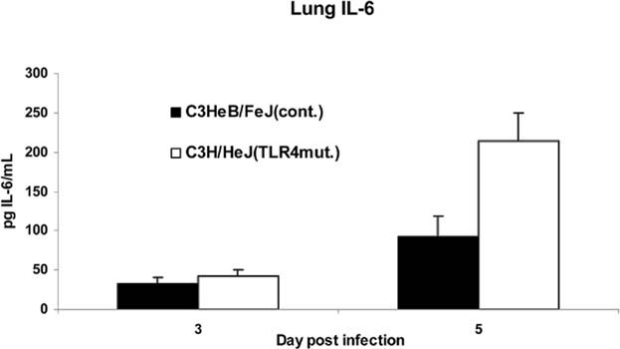
Lack of TLR4 does not impair proinflammatory cytokine production. IL-6 levels in the lung homogenate supernatants measured by ELISA. Error bars denote SEM. *p<0.05.

We hypothesized that protective effects of TLR4 may be mediated by pro-inflammatory cytokine responses, limiting viral replication and spread directly, or indirectly through recruitment of immune cells. To test this hypothesis, we infected C3H/HeJ and control mice with 5×10^5^ pfu Vac-GFL i.n. and harvested lungs on days 3 and 5 post-infection. Supernatants from homogenized lungs were analyzed by ELISA for IL-6, TNF-α, and MCP-1. There were no significant differences in levels of any of these cytokines between groups of mice on day 3. On day 5, TNF-α and MCP-1 levels were the same in TLR4 mutant and control mice, but IL-6 levels were significantly higher in C3H/HeJ lungs (Figure 8). No significant differences were detected in the plasma at either time (data not shown) (p>0.4). As with type I interferon, redundant signaling pathways are able to elicit NF-κB-dependent cytokine production in response to vaccinia infection.

To analyze the degree and composition of leukocyte infiltrates in the lung, immune cells were isolated from uninfected mice and mice on days 3 and 5 post-infection. Numbers and types of cells were analyzed by flow cytometry. At day 3, there was a trend towards higher total CD45+ cells in the C3H/HeJ cell, but these differences were not significant. We also measured subsets of immune cells in the lung, including B lymphocytes, CD4 and CD8 lymphocytes, macrophages, dendritic cells, and neutrophils. However, there were no consistent differences in cell types recruited to lungs of infected C3H/HeJ and control C3HeB/FeJ mice (data not shown).

**Figure 9:**
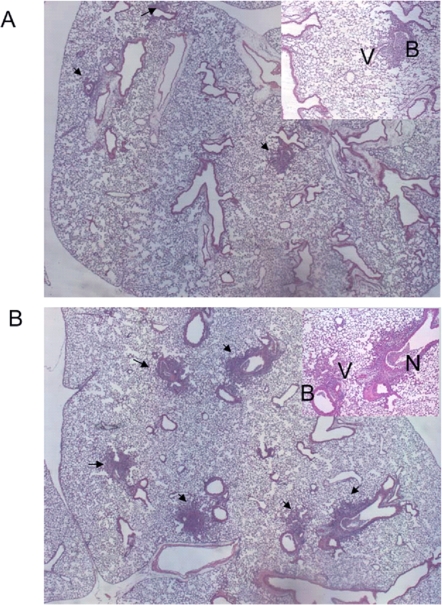
TLR4 alters the inflammatory response to vaccinia infection: representative photomicrographs. Mice were infected intranasally with 5×10^5^ pfu Vac-GFL. Lungs were harvested on days 3 and 5 post-infection, preserved in 10% formalin, paraffin-embedded, and stained with H&E. (A, B) representative sections of C3H/HeJ (A) and C3HeB/FeJ (B) lung tissue obtained on day 3 post-infection. B = bronchiole; V = blood vessel; arrows denote inflammatory foci.

**Figure 10:**
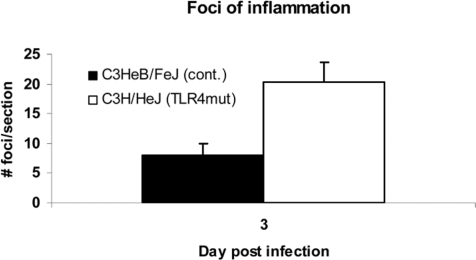
TLR4 alters the inflammatory response to vaccinia infection: quantification of foci of inflammation. Data are expressed as number of foci per section. *p<0.01. Error bars denote SEM.

To further assess the pattern of inflammation and tissue damage in TLR4 mutant lungs, we examined the lungs of vaccinia-infected mice by histology. Hematoxylin and eosin–stained sections showed foci of mixed and lymphocytic peribronchial and perivascular infiltrate (Figure 9A and 9B). Occasionally, infiltrating cells could be seen in alveoli separate from any peribronchial or perivascular focus. In foci of severe inflammation, epithelial cell necrosis was observed, and some inflammatory cells had apoptotic morphology. As a quantitative measure of inflammation, numbers of foci in each section were counted. On day 3 post-infection, the beginning of the interval when increased levels of virus could be discerned in lungs of TLR4 mutant mice, C3H/HeJ TLR4 mutant mice had significantly more foci of inflammation than controls (*p*<0.05; Figure 10). No consistent differences were detected on day 5. These data indicate that TLR4 is not required for producing an early local inflammatory response to vaccinia infection.

### Vaccinia predominantly infects epithelial cells in the lung

**Figure 11:**
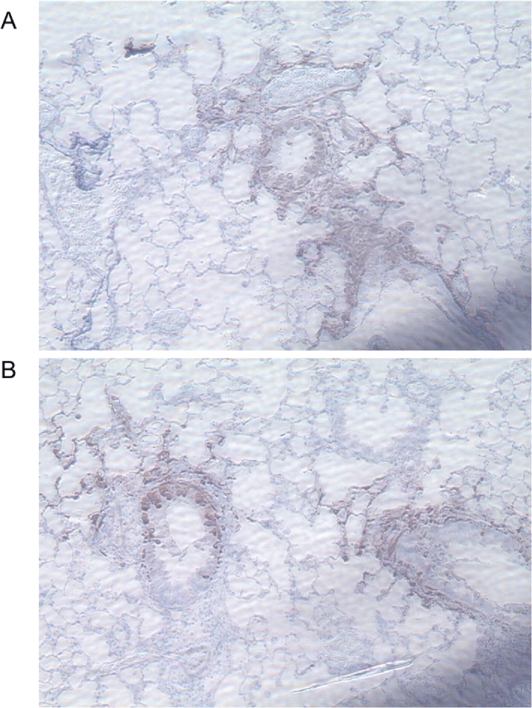
Vaccinia localizes to the bronchial and alveolar epithelium. Lungs harvested from Vac-GFL-infected mice (5×10^5^ pfu) on day 5 post-infection were stained with anti-GFP and counterstained with hematoxylin. (A) Representative section of C3H/HeJ lung, 150×. (B) Representative section of C3HeB/FeJ lung, 150×.

To investigate the cell type(s) involved in the propagation of infection in the lungs, we performed immunohistochemical staining on paraffin-embedded lung sections with anti-GFP. Mice were infected with 5×10^5^ pfu Vac-GFL, and lungs were harvested on days 3 and 5 post-infection. In all samples, intense anti-GFP staining was localized to bronchial epithelial cells with less extensive infection detected in alveolar epithelial cells (Figure 11A and 11B). Samples of both genotypes also showed some staining of cells among the inflammatory infiltrate, possibly macrophages, although firm identification could not be made. In all samples, the regions of positive anti-GFP antibody staining were associated with foci of inflammation, but many foci of inflammation had no regions of anti-GFP antibody staining. The distribution and types of infected cells did not differ between strains of mice. These findings suggest that in the lungs, vaccinia primarily replicates and spreads through epithelial cells.

### TLR4 recognizes a viral particle ligand

**Figure 12:**
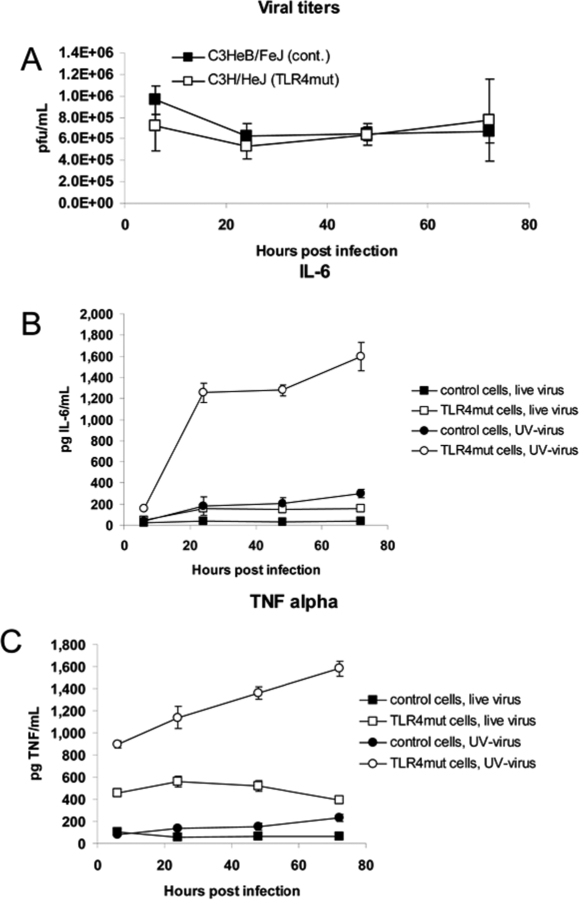
TLR4 recognizes an endogenous ligand and downregulates cytokine secretion. Control C3HeB/FeJ and TLR4 mutant C3H/HeJ bone marrow macrophages were treated with live or UV-inactivated Vac-GFL at MOI = 5 (5×10^5^ pfu). (A) Viral titers in macrophage cultures treated with live virus. IL-6 (B) and TNF-α (C) concentrations in supernatants measured by ELISA. For all combinations of pairs of points, *p*<0.05 except the pair denoting TNF-α in control cells treated with live vs. UV-inactivated virus at 6 hours post-infection.

In order to determine whether TLR4 was signaling in response to an endogenous or a viral ligand, we treated bone marrow macrophages isolated from C3H/HeJ and control mice with live or UV-inactivated virus (MOI = 5), and measured levels of TNF-α and IL-6 in the supernatant. The undiluted stock of UV-inactivated Vac-GFL (9.0×10^7^ pfu/mL) produced no plaques or cytopathic effect in cultured Vero cells (data not shown). TLR4 mutant and control macrophages were equally resistant to viral replication, even when challenged with live virus at a high MOI (Figure 12A). TLR4 mutant and control macrophages treated with UV-inactivated virus produced significantly (*p*<0.05) higher levels of IL-6 and TNF-α than cells of the same genotype treated with live virus (Figure 12B and 12C). This is likely due to the absence vaccinia-encoded inhibitors of TLR and other signaling pathways, such as N1L, A46R, and A52R [25]–[27], produced by replicating vaccinia virus. TLR4 mutant macrophages produced significantly higher levels of both IL-6 and TNF-α than control cells (*p*<0.05; both live and UV-inactivated virus). This shows that TLR4 not only is unnecessary for the cytokine response of bone marrow macrophages to vaccinia virus, but it actually dampens that response. C3H/HeJ (TLR4 mutant) cells treated with UV-inactivated virus produced by far the highest levels of any condition, rising above IL-6 levels in C3H/HeJ-with-live virus cultures by 6- to 7-fold and 2- to 3-fold for TNF-α in the same cells. Cytokine levels in UV-inactivated C3H/HeJ cultures were approximately 6- to 10-fold higher than those in UV-inactivated C3HeB/FeJ cultures. The fact that macrophages were able to produce TNF-α and IL-6 in response to UV-inactivated virus, and that TLR4-intact macrophages produce significantly less of these cytokines, indicates that neither viral replication nor cell death is necessary for TLR4 recognition of vaccinia virus. This suggests that TLR4 recognizes a component of the viral particle rather than an endogenous ligand released from infected host cells.

## Discussion

The innate immune system is vital for host defense against poxviruses, but molecular mechanisms of virus recognition and host defense are incompletely understood. While a robust Th1 immune response is necessary to eliminate vaccinia and other poxviruses, an exaggerated innate immune response also may threaten the life of the host. In septic shock, a systemic “cytokine storm” causes blood vessel dilation and activation of the clotting cascade, leading to hypotension, hemolysis, and multi-organ failure. Severe and fatal cases of smallpox are characterized by fever, hypotension, coagulopathy, blood vessel dilatation, and leukocyte extravasation, all of which are resemble the pathophysiology of septic shock [3]. These observations, coupled with the absence of lesions from any location except the skin, suggest that an uncontrolled systemic immune response is the most dangerous aspect of poxvirus infection.

As one mechanism through which immunity contributes to disease manifestations of poxviruses, we recently reported that TLR3 has a detrimental effect in vaccinia infection [13]. Specifically, mice lacking TLR3 had decreased viral replication, morbidity, and mortality following infection with vaccinia virus. These data established TLR3 as a key determinant of poxvirus pathogenesis and highlight the critical balance between effective and excessive innate immune responses during poxvirus infection.

To further investigate signaling pathways by which TLR3 exacerbates poxvirus disease, we first analyzed vaccinia infection in mice lacking TRIF, the only known adapter protein for TLR3. Unlike TLR3^−/−^ mice, viral replication was significantly greater in TRIF^−/−^ mice relative to wild-type animals. These data suggested the possibility that protective effects of TRIF against vaccinia infection were mediated through TLR4. Besides TLR3, TLR4 is the only other TLR known to use TRIF as a signaling adaptor. Although TLR4 canonically recognizes bacterial LPS, this receptor also has been implicated in host defense against some viruses. For example, TLR4 is reported to recognize respiratory syncytial virus (RSV) protein F or vesicular stomatitis virus (VSV) glycoprotein G, thereby initiating protective innate immune responses [11],[16].

Unlike TLR3, TLR4 does not rely on TRIF exclusively, but also can signal through the adaptor myeloid differentiation factor 88 (MyD88). We hypothesized that in TLR3^−/−^ mice, the normal inflammatory response was attenuated sufficiently to minimize injury to the host while still eliminating vaccinia virus. In TRIF^−/−^ mice, we reasoned that signaling inputs from TLR3 and TLR4 were both blocked, thus decreasing the inflammatory response to such a degree that the host was not able to make an effective defense against the virus. Consistent with this hypothesis, we demonstrated a protective effect for TLR4 in pulmonary vaccinia infection. Mice with an inactivating mutation in TLR4 suffered increased mortality, more severe hypothermia, and increased viral replication in the head, chest, and abdomen. Further investigation into the mechanism of this protection, however, revealed a more complicated picture.

The TLR4 signaling pathway results in activation of NF-κB and interferon regulatory factor 3, suggesting that TLR4-deficient mice would have increased viral replication because of impaired cytokine production and recruitment of immune cells to the lung and other sites of infection. However, levels of TNF-α, MCP-1, IL-6, and IFN-β in TLR4 mutant mice were equal to or even greater than those of the controls. Moreover, histological examination of infected lungs showed that TLR4 mutant mice had significantly more foci of inflammation in their lungs than did controls as early as day 3 post-infection. The results suggest either that TLR4 does not function in these aspects of host immunity to vaccinia virus or that other pattern recognition receptors compensate for loss of TLR4. For example, recent studies suggest protective functions of TLR2 and TLR9 in poxvirus infection [28],[29]. The fact that the TLR4 mutant mice are still more susceptible to disease indicates that other pattern recognition receptors are not fully redundant to TLR4 in poxvirus infection.

Immunohistochemical staining revealed vaccinia infection predominantly in bronchiolar epithelium with lesser amounts of viral GFP in alveolar epithelial cells. These data are consistent with previous studies showing that respiratory infection with poxviruses causes a necrotizing bronchopneumonia [30]. We also identified viral GFP antigen in immune cells in the lung, likely macrophages. Previous studies suggest that monocyte/ macrophage cell types are responsible for systemic spread of poxviruses [30]. While we cannot exclude the possibility that GFP is present in these cells because of phagocytosis rather than infection, our data are compatible with a model in which cells in the monocyte lineage are responsible for systemic dissemination of virus. The observation that both genotypes exhibited a similar repertoire of infected cells suggests that a difference in susceptibility of specific cell types does not account for increased susceptibility of the TLR4 mutant mice.

Increased IL-6 and TNF-α levels in TLR4 mutant macrophages treated with UV-inactivated virus show that viral replication and cell damage are dispensable for TLR4 recognition of vaccinia. This suggests that TLR4 recognizes a component of the viral particle rather than a host ligand. In our model, the ligand recognized by TLR4 likely would be located in/on the intracellular mature virion (IMV) particle, the predominant form of virus isolated by standard purification procedures such as those used in this research. TLR4 predominantly localizes to the cell membrane, so candidate TLR4 ligands likely would be on the surface of the intracellular mature virion. However, crosslinking DNA in the viral genome with UV/psoralen treatment does not prevent vaccinia from entering the cell and uncoating, so the TLR4 ligand also could be a capsid protein or another protein present in the viral particle.

Increased inflammation in TLR4 mutant mice may be secondary to increased viral burden or a primary effect of the loss of TLR4. Because TLR4 mutant macrophages secrete increased levels of IL-6 and TNF-α even when challenged with UV-inactivated virus, we propose that lack of TLR4 signaling causes increased inflammation. This interpretation also is supported by our data showing equal viral titers in TLR4 mutant and control macrophage cultures infected with live virus despite the higher cytokine levels in TLR4 mutant cell cultures. Consistent with our observations in TLR3^−/−^ mice, TLR4 may provide its protection by dampening the inflammatory response elicited in response to vaccinia infection.

In conclusion, this study demonstrates that TLR4 mediates a protective immune response to vaccinia virus. To our knowledge, it is the first to demonstrate such a role in the context of vaccinia infection, adding to a growing body of literature showing that TLR4 may respond to non-bacterial ligands and mediate protective effects against viruses. These data also highlight the complexity of TLR signaling in vivo in determining overall outcomes of infection. As the TLR4 mutant mice had equal or greater levels of interferon and proinflammatory cytokines in their lungs, we cannot attribute their increased susceptibility to impairment of TLR4-dependent interferon or cytokine production. Protective effects of TLR4 also cannot be attributed to altered or impaired effector cell recruitment or to increased susceptibility of a specific lung cell population to vaccinia infection. However, TLR4 differentially activates an aspect(s) of antiviral defense that is essential for early control of vaccinia replication and spread. Understanding details of this differential regulation will reveal strategies to enhance beneficial immunity to poxviruses and suppress detrimental host responses.

## Materials and Methods

### Mice

TRIF^−/−^ mice backcrossed to a C57BL/6 background were originally developed by the S. Akira laboratory and were bred at the University of Michigan. Wild-type (WT) C57BL/6J control mice were obtained from The Jackson Laboratory. Adult male and female mice ages 7 to 9 wk old were used for experiments. C3HeB/FeJ and C3H/HeJ mice were obtained from The Jackson Laboratory. Adult male mice ages 6 to 10 wk old were used for experiments. 17-wk-old mice were used as uninfected controls for histological studies.

### Vaccinia virus

We prepared stocks of Vac-GFL, a recombinant Western Reserve (WR) vaccinia virus that expresses firefly luciferase and GFP, and determined viral titers as described previously [13]. Viral titers in excised organs were analyzed by serial dilution on Vero cells [15].

### Cells

Vero cells were maintained as we previously have described [15]. Primary bone marrow macrophages were obtained by flushing the femurs and tibiae of mice with cold PBS. This suspension was filtered through a 100 µm filter and a 40 µm filter. Macrophages were cultured in Dulbecco's modified Eagle medium supplemented with 20% L929-cell-conditioned media, 10% heat-inactivated fetal bovine serum, 1% L-glutamine, and 0.1% penicillin-streptomycin. Macrophages were cultured 1 wk in this media before performing experiments with them.

### Animal procedures

All animal procedures were approved by the University of Michigan Committee on the Use and Care of Animals. Mice were infected i.n. with vaccinia virus as described previously [15]. Weights and rectal temperatures (Physitemp Instruments) were recorded on conscious mice immediately before infection and on each day throughout experiments.

### Bioluminescence imaging

Bioluminescence imaging was performed on each day after infection using an IVIS 200 system (Caliper). Imaging and data analysis were performed as described previously [15].

### Histology

To prepare lungs for histology, mice were euthanized on days 3 and 5 post-infection, and lungs were inflated with 1 mL of 10% formalin in PBS. Lungs were excised, preserved in 10% formalin overnight or longer, and then transferred to 70% ethanol solution. Fixed tissues were paraffin embedded and sectioned by the Morphology Core Facility at the University of Michigan. Tissue sections were stained with Gill's hematoxylin and counterstained with eosin. Sites of viral replication in the lungs were identified by immunohistochemistry, based on detection of GFP from Vac-GFL. Paraffin-embedded lung sections were stained using the Vector Laboratories ABC staining kit. Tissue sections were stained with rabbit polyclonal anti-GFP antibody (1/500 dilution) (Invitrogen) and goat anti-rabbit secondary antibody (1/200 dilution; Vector Laboratories). Blocking solution consisted a 1/67 dilution of goat serum in PBS with 250 mM total NaCl.

To quantify foci of inflammation in lung sections, we analyzed transverse lung sections through comparable portions of the upper and lower lobes of each lung. Sections were viewed under a 4× objective, and numbers of inflammatory foci were counted. Mean values for numbers of foci and SEM were calculated.

### Serum and tissue cytokines

Blood was obtained from the abdominal aorta of euthanized mice and collected into heparinized tubes. Plasma was separated from cells by centrifugation. Bronchoalveolar lavage of TRIF^−/−^ mice was performed by intratracheal instillation and withdrawal of 1 mL PBS in lungs of euthanized mice. Plasma and bronchoalveolar lavage fluid concentrations of TNF-α, IL-6, and MCP-1 were determined by ELISA performed by the University of Michigan Cancer Center Cellular Immunology Core Facility. Concentrations of IFN-β were measured by ELISA (PBL Biomedical Laboratories) according to the manufacturer's instructions.

Lungs were harvested on day 3 or 5 post-infection and homogenized in 5 mL PBS with a Polytron tissue homogenizer (Brinkmann). Lung homogenates were centrifuged at 2111×g for 10 minutes at 4°C. Supernatants were removed and concentrations of TNF-α, IL-6, and MCP-1 measured by ELISA as described above.

### Flow cytometry

Lungs were excised on day 3 or 5 post-infection and disaggregated by mechanical disruption in a blender (VWR). Cells were counted and analyzed by flow cytometry as described previously [31]. The following mAbs obtained from BD Pharmingen were used: RM4-4 (anti-murine CD4, rat IgG2b), 53-6.72 (anti-murine CD8, rat IgG2b), 1D3 (anti-murine CD19, rat IgG2a),M1/70 (anti-murine CD11b, rat IgG2b), HL3 (antimurine CD11c, hamster IgG1), 2.4G2 (anti-murine CD16/CD32 Fc block, rat IgG2b), 30-F11 (anti-murine CD45, rat IgG2b), and RB6-8C5 (anti-murine Ly6G Gr-1, rat IgG2b). Monoclonal Abs were primarily conjugated with FITC, PE, APC and APC-Cy7; biotinylated Abs were visualized using streptavidin-PerCP-Cy5.5 (BD Pharmingen). Isotype matched control mAbs (BD Pharmingen or eBioscience) were tested simultaneously in all experiments. All samples were analyzed on the BD LSR II flow cytometer with 3 lasers (488 nm blue, 405 nm violet and 633 nm HeNe red). CD45 APC-Cy7 and Invitrogen LIVE/DEAD Fixable Violet Dead Cell Stain were added to all lung mince samples. Subset analysis was performed on gated CD45-positive live cells. A minimum of 10,000 cells were analyzed for each sample. For all analyses, percentages for matched isotype control Abs were subtracted from values obtained for staining with specific Abs for individual markers.

### UV inactivation of virus

Vac-GFL was UV-inactivated by irradiation for 90 s on “sterilize” setting in a GS Genelinker UV-chamber (BioRad) following incubation in a Hank's Balanced Salt Solution (HBSS) solution containing 1.0 µg Psoralen according to the protocol of Puhlmann and colleagues [32].

### Statistics

Data were analyzed by *t* test for pairwise comparisons, using Microsoft Excel or GraphPad Prism software. Differences with *p*<0.05 were regarded as statistically significant.
